# Commentary: A Neural Basis for the Acquired Capacity for Suicide

**DOI:** 10.3389/fpsyt.2017.00093

**Published:** 2017-05-24

**Authors:** Xenia Gonda

**Affiliations:** ^1^Department of Psychiatry and Psychotherapy, Semmelweis University, Budapest, Hungary; ^2^MTA-SE Neuropsychopharmacology and Neurochemistry Research Group of the Hungarian Academy of Science, Semmelweis University, Budapest, Hungary; ^3^NAP-A-SE New Antidepressant Target Research Group, Semmelweis University, Budapest, Hungary; ^4^Laboratory for Suicide Prevention and Research, National Institute for Psychiatry and Addictions, Budapest, Hungary

**Keywords:** suicide, gender, neurobiology, imaging, completed suicide

Every year suicide accounts for nearly 2% or altogether one million deaths, one happening approximately every 40 s (1). Completed suicide happens significantly more frequently in males in all countries except for rural China (2), and in several countries, it is the leading cause of death in males younger than 40 years of age and is also among the leading causes in other age groups (3). In spite of this significant burden, our understanding of suicide is far from complete. Suicide is a complex phenomenon, arising in almost 90% in the framework of psychiatric disorders, mostly associated with affective illness. However, the lack of sufficient understanding of both psychosocial and neuroanatomical contributors and comprehensive biopsychosocial models leaves a huge gap in identifying targets for the early prediction, screening, detection, and intervention in case of suicidal behavior.

As mentioned earlier, completed suicide rates are much higher in men compared to women especially in the young age groups. Therefore, understanding the neurobiological background of the gender difference would be a promising approach for better understanding of the occurrence of this phenomenon and can also give biological support to psychosocial theories which in turn would also utilize biological findings. Although the most frequent precursor of suicide is major depression, which is three times more common in reproductive age women compared to men, males are paradoxically markedly overrepresented among suicide victims. Several clinical correlates of more common suicidal mortality can be identified in men including employment of more highly lethal methods, comorbid alcohol and substance use, less common help-seeking behavior, and increased impulsive aggressive traits (4). These are coupled with poorer treatment compliance and worse response to certain antidepressants in men further hindering recognition and prevention of processes leading to suicide in men (2). Therefore, identification of gender-specific factors in the emergence of suicide needs to be identified to design-specific interventions. Furthermore, understanding the role of gender in differences in the manifestation of behaviors along the suicide spectrum would also yield important new insight concerning the nature of suicide in general.

There are important gender differences in brain processes involved in learning, memory, language, fear, and anxiety as well as significant gender differences in the prevalence rates of several neuropsychiatric disorders, which may result from sex chromosome effects, developmental hormonal effects, or hormonal modulations during adulthood (5) and may also be consequences of gender-related psychosocial effects. So far, gender differences in dopamine and serotonin functions and genetics (6) and associations between cortisol and male suicide have been reported (7, 8). Testosterone also plays an important role in the gender effect observable in suicide, and this effect may also interact with age (9). Urocortin and BDNF also appear to play a gender-specific role in male suicidal behavior (10), and interestingly there appears to be a gender difference in the putative antisuicidal effect of lithium as well (11). This already complex picture suggests that the investigation of genetic and biological factors in the background of suicide should be conducted from a gender perspective.

In line with this previous knowledge, the present paper (12) focuses on understanding the neural substrates in the background of gender differences in completed suicide by investigating a potential neural network for acquired capability for suicide (ACS), which includes emotional stoicism, sensation-seeking, pain tolerance, fearlessness of death, thus involving neurobiological structures and processes associated with various endophenotypes of suicidal behavior outlined using activation likelihood estimation meta-analysis. The authors report that male-specific neural ACS networks are both more widespread and more diverse compared to female-specific networks and are dominated by motor regions, while female networks show a dominance of limbic regions, explaining why suicidal behavior is manifested more on the action and motor level in males and on the emotional and ideation level in females.

One important aspect of this paper is that while most imaging studies report on suicide attempts, this is one of the rare studies focusing on imaging methodology in completed suicide. As gender shows an inverse association with attempted vs. completed suicide, studies on suicide attempters are unable to address the question of gender sufficiently. Furthermore, the current study uses a psychosocial theory and psychological constructs as a basis for neurobiological research for a comprehensive approach. The results also validate the construct of ACS which may be a key and potentially operationalizable construct in suicidal behavior, by identifying its underlying neural substrates.

This research indeed provides us with important new data on the gender differences in completed suicide; however, the applicability of its method and approach goes way beyond that. Rather than focusing on suicidal behavior as a single entity, or on suicide-related single individual endophenotypes such as impulsiveness or hopelessness, this paper with a unique novel approach targets a complex pathway of behaviors leading to the emerging risk for suicide. Thus, these novel results not only provide a new insight into the gender differences in fatal suicides but also set an example to the complex approach of this complex phenomenon, which may pave the way for the development of more detailed and accurate dynamic models in the understanding of suicidal behavior helping to identify targets for prediction and intervention in both genders.

## Author Contributions

XG undertook a review of the literature, conceived this general commentary, and wrote and reviewed all drafts.

## Conflict of Interest Statement

The author declares that the research was conducted in the absence of any commercial or financial relationships that could be construed as a potential conflict of interest.
